# ANP32 Family as Diagnostic, Prognostic, and Therapeutic Biomarker Related to Immune Infiltrates in Hepatocellular Carcinoma

**DOI:** 10.1155/2022/5791471

**Published:** 2022-03-03

**Authors:** Xuxu Liu, Yuanhang He, Pengfei Wang, Jie Hu, Chenjun Hao, Qiang Wang, Yang Yang, Yuanyuan Sun, Biao Ma, Hezheng Sun, Dongbo Xue, Xianzhi Meng

**Affiliations:** ^1^Department of General Surgery, The First Affiliated Hospital of Harbin Medical University, Harbin, Heilongjiang, China; ^2^Key Laboratory of Hepatosplenic Surgery, Ministry of Education, The First Affiliated Hospital of Harbin Medical University, Harbin, Heilongjiang, China; ^3^Department of Centric Operating Room, The First Affiliated Hospital of Harbin Medical University, Harbin, Heilongjiang, China; ^4^Department of General Surgery, The Fourth Affiliated Hospital of Harbin Medical University, Harbin, Heilongjiang, China

## Abstract

Hepatocellular carcinoma (HCC) is one of the most common tumors worldwide, with high incidence and mortality rate. There is an urgent need to identify effective diagnostic and prognostic biomarkers for HCC. Members of the acidic leucine-rich nucleophosphoprotein 32 (ANP32) family, which mainly includes *ANP32A*, *ANP32B*, and *ANP32E*, are abnormally expressed and have prognostic value in certain cancers. However, the diagnostic, prognostic, and therapeutic value of ANP32 family members in HCC has not yet been fully studied. In this study, we identified the diagnostic and prognostic value of ANP32 family members in HCC. Transcriptome data from public databases, such as the Cancer Genome Atlas (TCGA) and Genotype-Tissue Expression (GTEx) databases, suggested that *ANP32A*, *ANP32B*, and *ANP32E* were upregulated in HCC tissues, and high expression of ANP32 family members was associated with advanced pathologic stage and histologic grade. Our immunohistochemistry and western blot results further verified the differential expression of ANP32 family members. *ANP32A*, *ANP32B*, and *ANP32E* had an outstanding diagnostic potential. Survival analysis of HCC patients in TCGA databases demonstrated that *ANP32A*, *ANP32B*, and *ANP32E* were associated with poor overall survival (OS) and disease-specific survival (DSS). Univariate and multivariate Cox analyses suggested the capability of *ANP32B* and *ANP32E* to independently predict the OS and DSS of HCC patients. Gene set enrichment analysis (GSEA) showed that ANP32 family members were associated with immune response, epidermal cell differentiation, and stem cell proliferation. Expression of ANP32 family members was associated with immune cell infiltration and immune status in the tumor microenvironment of HCC, and patients with high ANP32 family expression had poor sensitivity to immunotherapy. Finally, we identified potential chemotherapy drugs for HCC patients with high ANP32 family expression by CellMiner database. This study suggested the diagnostic, prognostic, and therapeutic roles of the ANP32 family in HCC patients, providing potential therapeutic targets for HCC.

## 1. Introduction

Hepatocellular carcinoma (HCC), a highly heterogeneous tumor, is among the top five cancers with the highest mortality rates [1, 2]. The occurrence of HCC is usually related to hepatitis B or C virus infection, alcohol, and aflatoxin [3]. Most HCC patients are already at an advanced stage at initial diagnosis, accounting for their poor prognoses. The mortality rate of HCC among all cancers increased from third in 2018 to second in 2020 [4]. Due to its high morbidity and mortality, HCC has received increasing attention. Thus, new biomarkers for the early diagnosis, treatment, and prognosis of HCC are urgently needed.

The acidic leucine-rich nucleophosphoprotein 32 (ANP32) family is a class of highly conserved proteins characterized by a leucine-rich repeat sequence (LRR) in the N-terminus and a low-complexity acidic region (LCAR) in the C-terminus rich in aspartic acid and glutamic acid [5]. A previous review concluded that there are eight members of the ANP32 family (*ANP32A*-*H*) [6]. However, only *ANP32A*, *ANP32B*, and *ANP32E* have been isolated at the transcription and protein levels multiple times in mammals [7]. *ANP32C* and *ANP32D* have been described as pseudogenes, and other members of the ANP32 family (*ANP32F, ANP32G,* and *ANP32H*) lack sufficient evidence to support their transcription and translation [7]. Therefore, we decided to adhere to the view of Reilly et al. that only *ANP32A*, *ANP32B*, and *ANP32E* can truly be considered members of the ANP32 family in mammals [7]. Members of the ANP32 family participate in various molecular biological processes, such as embryonic development, chromatin modification and reconstruction, and apoptosis, by regulating cell signals and gene expression [7–9]. Many studies have shown that members of the ANP32 family are differentially expressed in certain cancers, such as pancreatic cancer, prostate cancer, breast cancer, glioblastoma, and leukemia, playing tumor-suppressive and oncogenic roles [10–12]. ANP32 family members can also play prognostic roles in certain tumors. High *ANP32A* expression indicates poor prognosis of acute myeloid leukemia and glioma [13, 14]. Patients with high expression of *ANP32B* and *ANP32E* had poor prognosis in breast cancer [15, 16]. A study by Tian et al. showed that *ANP32A* not only promotes the progression of HCC but also indicates a poor prognosis of HCC [17]. However, this research did not conduct a more systematic analysis of the prognostic role of *ANP32A* in HCC. In addition, the diagnostic, prognostic, and therapeutic value of *ANP32B* and *ANP32E* in HCC has not been studied.

In this research, we explored the diagnostic, prognostic, and therapeutic value of ANP32 family members in HCC with systematic bioinformatics methods, providing potential therapeutic targets for HCC patients.

## 2. Materials and Methods

### 2.1. Data Sources

The RNA-seq data (FPKM format) and clinical information of 374 HCC samples in the TCGA-LIHC project were derived from the UCSC Xena link (http://www.genome.ucsc.edu/index.html). RNA-seq data of the normal liver samples included paracancerous tissue in TCGA-LIHC (*n* = 50) and normal liver tissue in GTEx (*n* = 110), and the datasets for which were also downloaded from the UCSC Xena data center. Microarray transcriptome data came from the TNMplot online tool (https://tnmplot.com/analysis/), which contains 379 normal liver tissues, 806 primary HCC tissues, and 24 metastatic HCC tissues [18].

### 2.2. Differential Expression Analysis of the ANP32 Family in HCC

HCC samples and paracancerous samples in TCGA and GTEx were used for differential analysis of the ANP32 family. TNMplot was used to investigate the differential expression of ANP32 family members in normal liver tissues, primary HCC tissues, and metastatic HCC tissues. The potential of the ANP32 family to differentiate HCC tissues versus normal tissues was identified by receiver operating characteristic (ROC) curve analysis and area under the curve (AUC).

### 2.3. Survival Analysis

Prognostic indicators included overall survival (OS) and disease-specific survival (DSS). *ANP32A*/B/E was divided into high and low expression groups according to the median value of expression. Comparison of prognosis between the high and low expression groups was completed by Kaplan–Meier survival analysis. Univariable and multivariable Cox analyses were employed to identify independent prognostic factors, and only significant factors on univariate Cox analysis (*P* ≤ 0.05) were selected for multivariate Cox analysis.

### 2.4. Protein–Protein Interaction (PPI) Network

GeneMANIA (http://genemania.org) is a user-friendly online database that allows researchers to explore the functions and interactions between genes or gene sets of interest [19]. A total of 660,554,667 interactions and 166,691 genes of 9 species are contained in GeneMANIA. In this study, we explored proteins that interact with ANP32 family members in Homo sapiens and constructed a PPI network through GeneMANIA.

### 2.5. Gene Set Enrichment Analysis (GSEA)

First, the “gmt” file of the c2 Reactome gene set and the c5 Gene Ontology (GO) gene set were downloaded from the MSigDB database (https://www.gsea-msigdb.org/gsea/msigdb/index.jsp). c2 Reactome contains canonical pathway gene sets derived from the Reactome pathway database, while c5 GO contains gene sets derived from Gene Ontology. Then, GSEA based on c2 Reactome and c5 GO was performed by the clusterProfiler package in R software (significance thresholds: *P* value < 0.05 and *q* value < 0.25).

### 2.6. Immune Infiltration Analysis

A single-sample GSEA (ssGSEA) algorithm was performed to evaluate the 24 immune cell populations and 14 immune statuses. The relationship between immune subtypes and ANP32 family member expression was analyzed and visualized by the TISIDB database (http://cis.hku.hk/TISIDB) [20].

### 2.7. Drug Sensitivity Analysis

We used the Tumor Immune Dysfunction and Exclusion (TIDE) algorithm to assess the sensitivity of HCC patients to immune checkpoint blockers (ICBs) in the TCGA cohort [21]. Generally, patients with high TIDE scores are less sensitive to ICB treatment [21]. The correlation between ANP32 family member expression and drug response was predicted by CellMiner [22]. We selected the top 16 drugs approved by the FDA with the strongest positive correlation coefficient between sensitivity and expression as candidate drugs (*P* < 0.05).

### 2.8. cBioPortal Database

cBioPortal (http://www.cbioportal.org) is an open-source web portal that can be used to explore genetic alterations such as mutation and copy number variation (CNV) [23]. We used cBioPortal to explore the relationship between genetic alterations of ANP32 family members and the prognosis of HCC patients in selected studies.

### 2.9. Immunohistochemistry (IHC)

We obtained HCC tissues (*n* = 5) and paracancerous tissues (*n* = 5) from the First Affiliated Hospital of Harbin Medical University. *ANP32A* antibody (DF13532) was purchased from Affinity Biosciences. *ANP32B* antibody (CY8229) was purchased from Abways Technology. *ANP32E* antibody (ab5993) was purchased from abcam. The paraffin sections were deparaffinized, the endogenous enzymes were inactivated, and the antigens were thermally repaired. The sections were then blocked and stained with antibodies against *ANP32A*, *ANP32B*, and *ANP32E* (dilution 1 : 100), followed by the corresponding secondary antibody and a Streptavidin Biotin Complex kit (Boster BioEngineering, Wuhan, China). The stained slides were scanned by Panoramic SCAN (3DHISTECH Kft, Budapest, Hungary).

### 2.10. Western Blot

HCC tissues (*n* = 3) and paracancerous tissues (*n* = 3) obtained from the First Affiliated Hospital of Harbin Medical University were gently washed three times with PBS, and the tissues were lysed with RIPA buffer to extract total protein. Equal amounts of protein samples were separated on a 10% polyacrylamide gel and then transferred to a PVDF membrane. After blocking with 5% skimmed milk, the membrane was incubated with *ANP32A* (Affinity, DF13532), *ANP32B* (Abways, CY8229), and *ANP32E* (Abcam, ab5993) antibodies (both dilutions 1 : 1000) overnight in a refrigerator at 4°C and then rinsed with PBST (phosphate-buffered saline with 0.1% Tween 20) three times for 10 minutes each. The secondary antibody was incubated at room temperature for 1 h and then washed 3 times with PBST. GAPDH was used as a control. The Odyssey CLx Imaging System (LI-COR Biosciences, USA) was used for scanning, and Image Studio software was used to analyze the gray values of the images.

### 2.11. Statistical Analysis

Paired-sample Student's *t* test was used for comparisons between paired samples. For the comparison between the two groups of data, the *t* test or the Mann–Whitney *U* test was selected according to whether the data obeyed the normal distribution. The chi-square test or Fisher's exact test was used for categorical data. The Kruskal–Wallis test was used for comparisons of more than two groups. All correlation analyses adopted Spearman analysis. Survival analysis adopted Kaplan–Meier survival analysis and the log-rank test. All statistical analyses were performed in R, version 4.0.2, and *P* < 0.05 indicates a statistically significant difference (^∗^*P* < 0.05, ^∗∗^*P* < 0.005, and ^∗∗∗^*P* < 0.001; ns: *P* > 0.05).

## 3. Results

### 3.1. ANP32 Family Members Were Ppregulated in HCC

**Figure 1:**
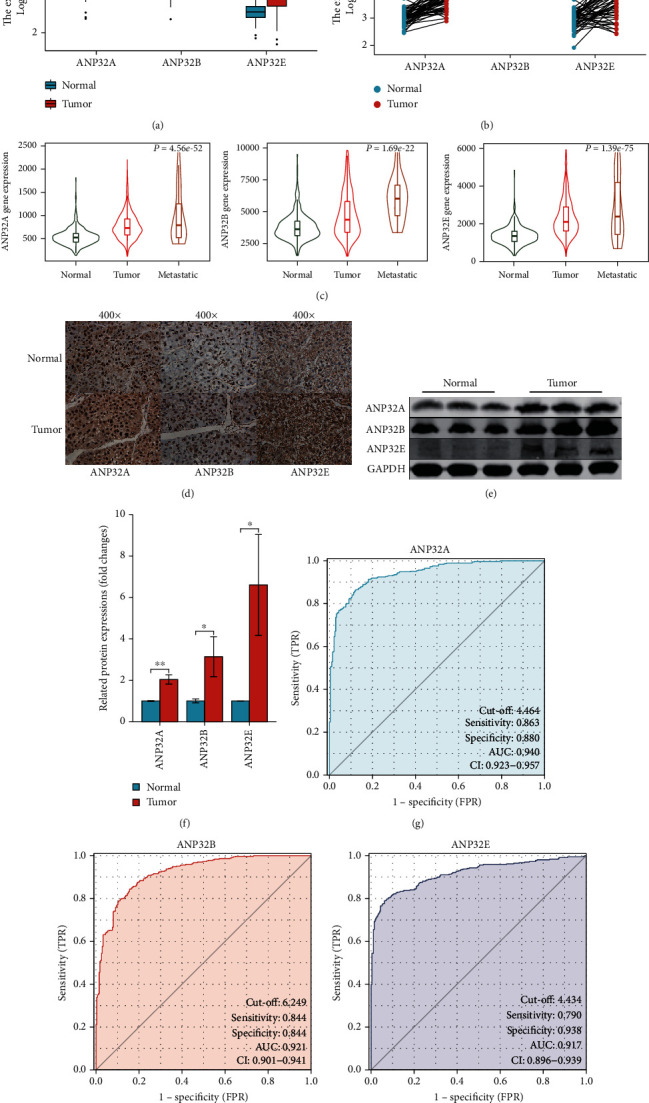
Expression and diagnostic value of ANP32 family members in hepatocellular carcinoma (HCC). (a) Differential expression of ANP32 family members between HCC tissues and normal liver tissues in the TCGA + GETx cohort. (b) Differential expression of ANP32 family members between HCC tissues and normal liver tissues in paired samples from the TCGA cohort. (c) Expression of ANP32 family members in normal liver tissues, primary HCC tissues, and metastatic HCC tissues by TNM plot. (d) Expression of ANP32 family members in HCC tissues and normal liver tissues by immunohistochemistry. (e) Expression of ANP32 family members in HCC tissues and normal liver tissues by western blot. (f) Quantification of western blot data. (g)–(i) Diagnostic ROC curves of *ANP32A* (g), *ANP32B* (h), and *ANP32E* (i) in the TCGA + GETx cohort.

The combined analysis of the TCGA and GTEx databases showed that compared with normal liver tissues, *ANP32A*, *ANP32B*, and *ANP32E* were expressed at significantly higher levels in the HCC samples (Figure 1(a)). Difference analysis between paired samples showed the same results (Figure 1(b)). The results from TNM plot tools demonstrated that the expression levels of *ANP32A*, *ANP32B*, and *ANP32E* were higher in metastatic HCC tissue than in primary HCC and normal tissue (Figure 1(c)). IHC showed that compared with paracancerous tissue, *ANP32A*, *ANP32B*, and *ANP32E* were all highly expressed in HCC (Figure 1(d)). Western blot analysis further verified the differential expression of the ANP32 family at the protein level. The western blot results in Figures 1(e) and 1(f) show that the expression levels of *ANP32A*, *ANP32B*, and *ANP32E* in HCC were higher than those in adjacent tissues.

The ROC curve showed that *ANP32A*, *ANP32B*, and *ANP32E* had strong capabilities for identifying HCC samples and normal liver samples (Figures 1(g)–1(i)). The AUCs were 0.940 for *ANP32A* (Figure 1(g)), 0.921 for *ANP32B* (Figure 1(h)), and 0.917 for *ANP32E* (Figure 1(i)).

### 3.2. Expression of ANP32 Family Members and Clinical Characteristics of HCC Patients

Tables 1, 2, and 3 show the clinical characteristics of 374 HCC patients in TCGA and their relationship with *ANP32A*, *ANP32B*, and *ANP32E* expression, respectively.  Table 1 shows that compared with patients in the *ANP32A*-low expression group, the *ANP32A*-high expression group had more female patients, younger patients (≤60 years), and low-height patients (<170 cm) (all *P* < 0.05). Importantly, high *ANP32A* expression was related to vascular invasion, higher histologic grade, and higher blood AFP concentration (>400 ng/ml) (all *P* < 0.05).  Table 2 suggests that compared with *ANP32B*-low expression, *ANP32B*-high expression was associated with lower weight (≤70 kg), height, and BMI (≤25) but higher T stage, pathologic stage, histologic grade, blood AFP concentration and tumor status (all *P* < 0.05). Compared with the *ANP32E*-low expression group, the *ANP32E*-high expression group was related to lower weight but higher histologic grade and AFP concentration (Table 3, all *P* < 0.05). In addition, patients in the *ANP32E*-high expression group tended to have higher T stage (*P* = 0.056) and pathologic stage (*P* = 0.055).

### 3.3. ANP32 Family Members Were Associated with the Progression and Metastasis of HCC

**Figure 2:**
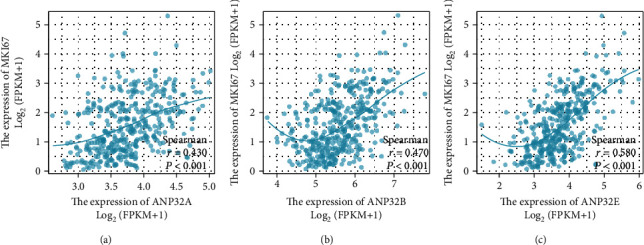
ANP32 family member expression was related to *Ki-67*. Relationships between Ki-67 and *ANP32A* (a), *ANP32B* (b), and *ANP32E* (c).

Our results indicated that the ANP32 family may be associated with HCC progression and metastasis (Figure 1(c) and Tables 1, 2, and 3). Therefore, we explored the correlation between ANP32 family members and the cell proliferation marker *Ki-67 (MKI67)* as well as the invasion marker vimentin (VIM). The results showed that the expression levels of *ANP32A*, *ANP32B*, and *ANP32E* showed a strong correlation with the expression of *Ki-67* (Figure 2).

### 3.4. Prognostic Value of ANP32 Family Members in HCC Patients

**Figure 3:**
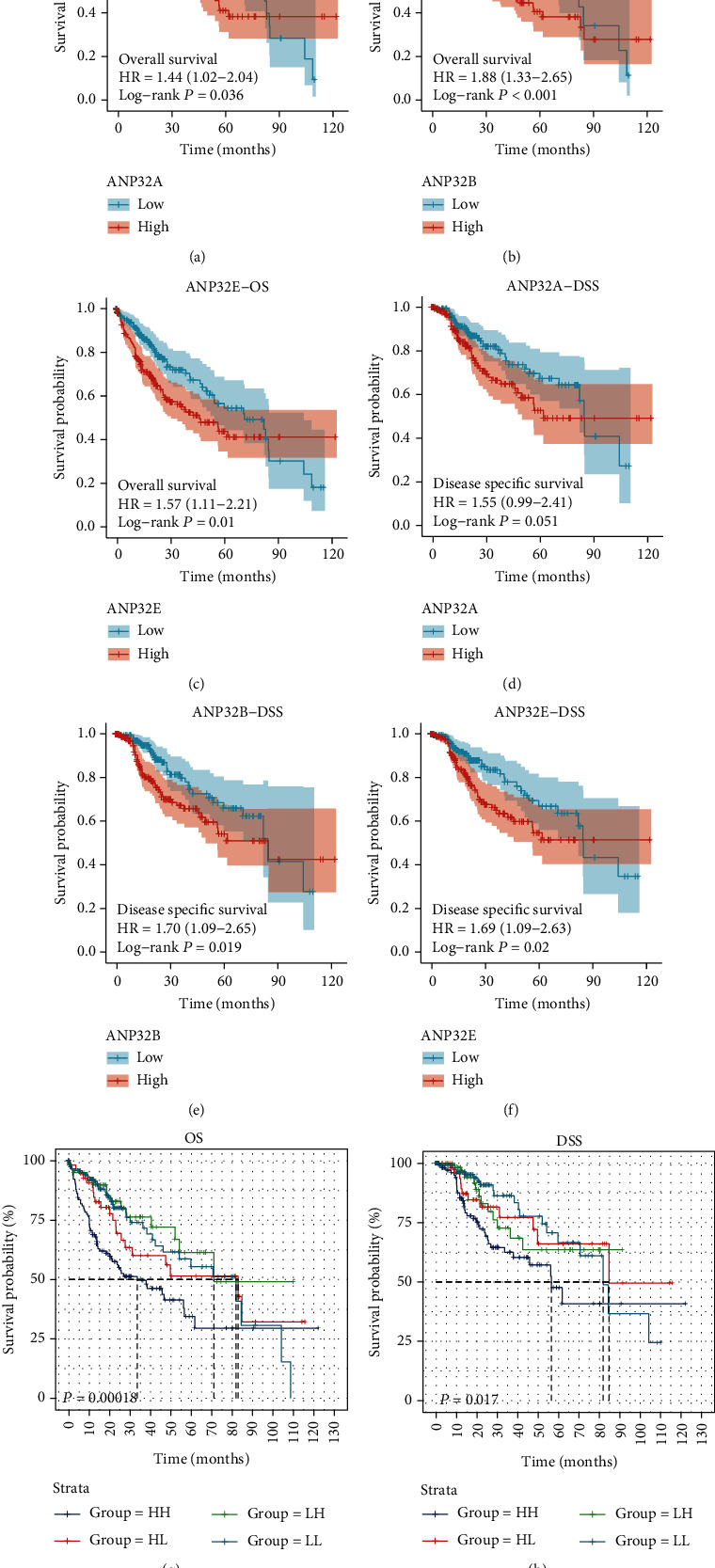
Prognostic value of ANP32 family members in HCC. (a)–(c) Association between the expression of ANP32 family members and overall survival (OS). (d)-(f) Association between the expression of ANP32 family members and disease-specific survival (DSS). (g) Combination of *ANP32B* and *ANP32E* for OS in HCC patients. (h) Combination of *ANP32B* and *ANP32E* for DSS in HCC patients.

HCC patients in the *ANP32A*/B/E-high expression group had strikingly worse OS than those in the *ANP32A*/B/E-low expression group (Figures 3(a)–3(c)) (all *P* < 0.05). Patients in the *ANP32A*-high expression group tended to have worse DSS than those in the *ANP32A*-low expression group, but the difference was not statistically significant (Figure 3(d)) (*P* = 0.051). High expression of *ANP32B*/E was significantly associated with shorter DSS (Figures 3(e) and 3(f)).

To further determine whether ANP32 family expression served as an independent variable for the OS and DSS of HCC patients, we performed univariate and multivariate Cox analyses. Only significant variables on univariate Cox analysis (*P* ≤ 0.05) were selected for multivariate Cox analysis. The results suggested that *ANP32B* and *ANP32E* can be used as independent predictors of both OS (Table 4) and DSS (Table 5).

Subsequently, *ANP32B* and *ANP32E* were combined to predict the prognosis of HCC patients. Based on the median value of the expression of *ANP32B* and *ANP32E*, we divided the HCC patients into an HH subgroup (high expression levels of both *ANP32B* and *ANP32E*), an HL subgroup (high expression of *ANP32B* but low expression of *ANP32E*), an LH subgroup (low expression of *ANP32B* but high expression of *ANP32E*), and an LL subgroup (low expression levels of both *ANP32B* and *ANP32E*). As shown in Figures 3(g) and 3(h), the OS and DSS of the HH subgroup were worse than those of the other subgroups (all *P* < 0.05). This indicated that the combination of *ANP32B* and *ANP32E* provided more precise information for prognosis.

### 3.5. Protein-Protein Interaction (PPI) Network and GSEA

**Figure 4:**
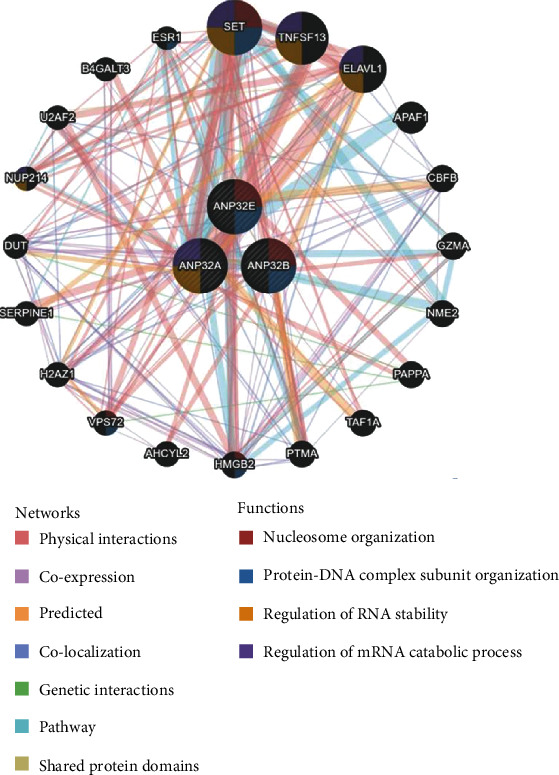
Protein-protein interaction (PPI) network of the ANP32 family.

To explore the proteins that interact with ANP32 family members, we used GeneMANIA to construct and visualize PPI networks. Among the 20 proteins that interact with the ANP32 family, SET, TNFSF13, ELAVL1, and APAF1 are the most closely related proteins. The functions of ANP32 family members and these proteins are mainly related to processes such as nucleosome organization, protein-DNA complex subunit organization, regulation of RNA stability, and regulation of the mRNA catabolic process (Figure 4).

**Figure 5:**
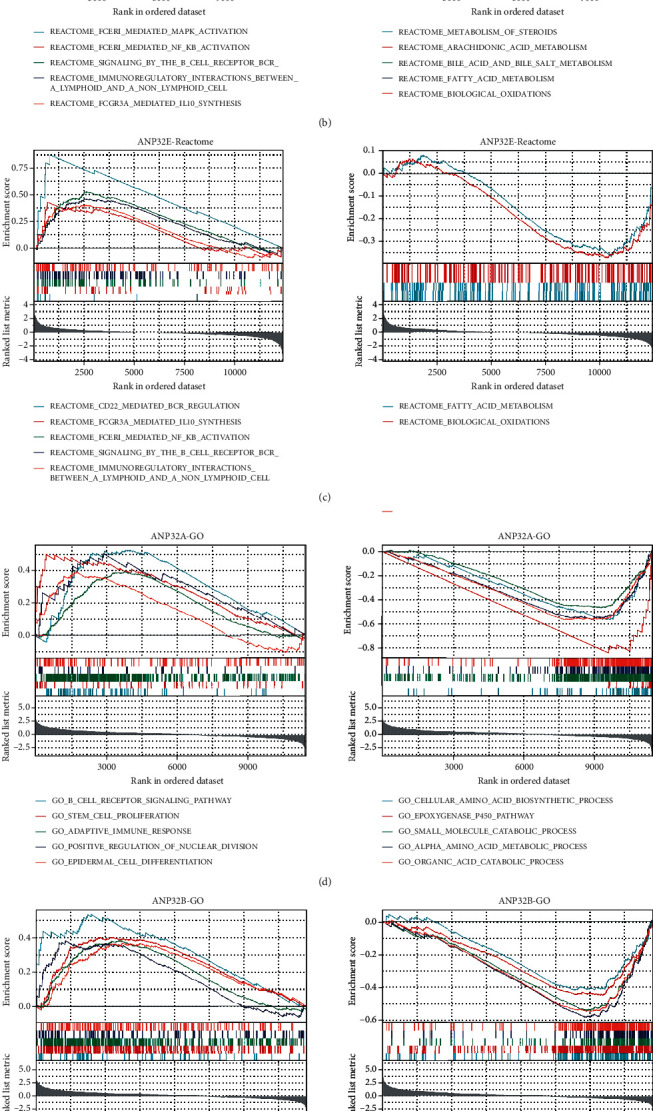
Gene set enrichment analysis (GSEA) of the ANP32 family. (a) and (b) GSEA for *ANP32A* based on Reactome pathways and GO. (c) and (d) GSEA for *ANP32B* based on Reactome pathways and GO. (e) and (f) GSEA for *ANP32E* based on Reactome pathways and GO.

GSEA based on the Reactome pathways showed that *ANP32A*, *ANP32B*, and *ANP32E* were positively associated with immune response-related pathways such as FCERI-mediated NF-*κ*B activation, signaling by the B cell receptor (BCR), FCERI-mediated MAPK activation, and FCGR3A-mediated IL10 synthesis (Figures 5(a)–5(c)). GSEA based on GO suggested that ANP32 family members were positively associated with immune response-related processes (Figures 5(d)–5(f)). In addition, *ANP32A* and *ANP32B* were positively associated with stem cell proliferation and epidermal cell differentiation (Figures 5(d) and 5(e)). Both GSEA based on Reactome and GO suggested that ANP32 family members were negatively associated with metabolism-related pathways such as biological oxidations and fatty acid metabolism (Figures 5(a)–5(f)).

### 3.6. Relationship between ANP32 Family Members and Immune Cell Infiltration

**Figure 6:**
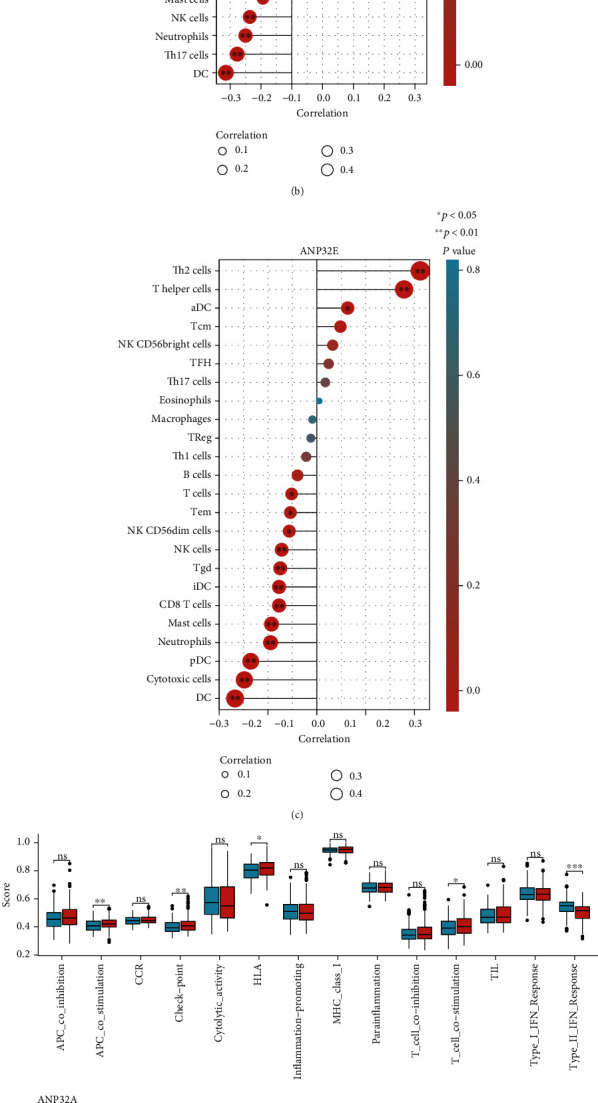
Relationships between ANP32 family members and immune characteristics. (a)–(c) Relationship between ANP32 family member expression and immune cell infiltration by ssGSEA. (d)–(f) Relationship between ANP32 family member expression and immune status by ssGSEA. (g) Associations between *ANP32A*, *ANP32B*, and *ANP32E* with immune subtypes in HCC by TISIDB.

Since functional enrichment analysis showed that ANP32 family members are related to the immune response, we used the ssGSEA algorithm to explore the relationship between the ANP32 family and immune cell infiltration as well as immune status in the HCC tumor microenvironment. We found that *ANP32A* expression was positively correlated with the infiltration of Th2 cells, NK CD56bright cells, and TFH (T follicular helper) cells but negatively correlated with Treg cells, Tcm (T central memory) cells, cytotoxic cells, DCs, TGD (T gamma delta) cells, Th17 cells, and neutrophils (Figure 6(a)). *ANP32B* expression was positively correlated with Th2 cells, TFH cells, NK CD56bright cells, Th1 cells, T helper cells, aDCs (activated DCs), and NK CD56dim cells and negatively correlated with NK cells, neutrophils, DCs, and Th17 cells (Figure 6(b)). *ANP32E* expression was positively associated with Th2 cells, T helper cells, and aDCs but negatively associated with T cells, Tem (T effector memory) cells, NK CD56dim cells, NK cells, Tgd, iDCs (emotional DCs), CD8 T cells, mast cells, neutrophils, pDCs (plasmacytoid DCs), cytotoxic cells, and DCs (Figure 6(c)).

Compared with the *ANP32A* low expression group, the *ANP32A* high expression group had higher APC costimulation, checkpoint, HLA, and T cell costimulation scores but lower Type II IFN response scores (Figure 6(d)). High *ANP32B* expression was positively correlated with checkpoint scores but negatively correlated with type I IFN response and type II IFN response scores (Figure 6(e)). High *ANP32E* expression was positively associated with MHC class I scores but negatively correlated with cytolytic activity and type I IFN response and type II IFN response scores (Figure 6(f)).

Subsequently, we explored the relationship between the expression of ANP32 family members and the immune subtypes of HCC in the TISIDB database. The available immune subtypes included C1 to C6 (C1: wound healing, C2: IFN-gamma dominant, C3: inflammatory, C4: lymphocyte depleted, C5: immunologically quiet, and C6: TGF-b dominant) [24]. We found that *ANP32A*, *ANP32B*, and *ANP32E* were highly expressed in the C1 and C2 subtypes and expressed at low levels in the C3 subtype (Figure 6(g)).

### 3.7. ANP32 Family Members and Drug Treatment Sensitivity

**Figure 7:**
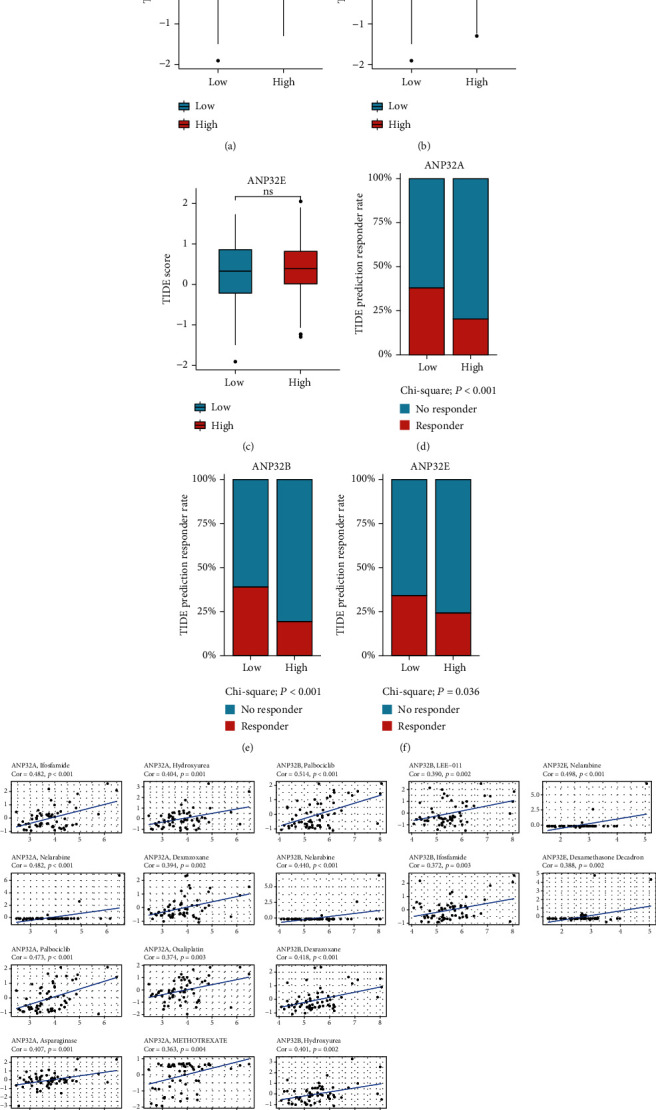
Relationship between ANP32 family members and immune checkpoint inhibitor (ICB) therapy sensitivity. (a)–(c) Relationship between ANP32 family member expression and TIDE score. (d)–(f) Relationship between ANP32 family member expression and the responders of HCC patients to ICB treatment. (g) Relationship between ANP32 family members and chemosensitivity to selected drugs.

Immune checkpoint inhibitor (ICB) therapy has played a landmark role in the treatment of HCC in recent years [25]. However, only a few patients can benefit from it, and there are still a great number of individuals who do not respond to ICB therapy. Therefore, we evaluated the relationship between ANP32 family members and ICB treatment sensitivity. As shown in Figures 7(a)–7(c), *ANP32A* and *ANP32B* were associated with higher TIDE scores, while *ANP32E* had no correlation with TIDE scores. The TIDE algorithm can predict the response of patients to immunotherapy. Compared with the *ANP32A*/B/E-high expression group, the *ANP32A*/B/E-low expression group had a higher proportion of patients who responded to immunotherapy (all *P* < 0.05).

To identify chemotherapy drugs to which patients with high expression of ANP32 family members are sensitive, we explored the CellMiner database to find drugs whose sensitivity was significantly positively correlated with ANP32 family expression (Cor > 0.3) among the drugs approved by the FDA. As shown in Figure 7(g), we identified 16 drugs to which patients with high ANP32 family expression were sensitive. Among them, the drugs whose sensitivity was significantly positively correlated with *ANP32A* expression were palbociclib, ifosfamide, nelarabine, asparaginase, hydroxyurea, dexrazoxane, oxaliplatin, and methotrexate. The drugs whose sensitivity was significantly positively correlated with *ANP32B* expression were palbociclib, nelarabine, dexrazoxane, hydroxyurea, LEE−011, and ifosfamide. The drugs whose sensitivity was strikingly positively associated with *ANP32E* expression were nelarabine and dexamethasone.

### 3.8. Genetic Alterations of ANP32 Family Members in HCC

**Figure 8:**
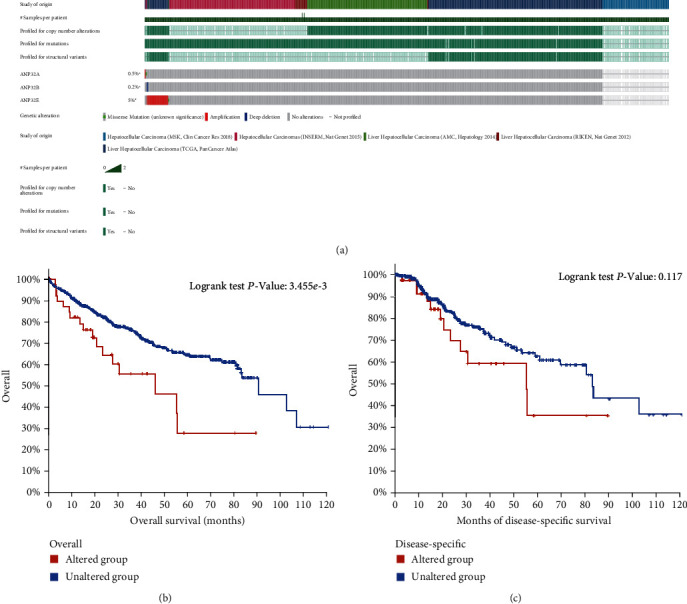
Genetic alterations of ANP32 family members in HCC. (a) ANP32 family member mutation in HCC. (b) Association between ANP32 family member mutations and OS in HCC patients. (c) Association between ANP32 family member mutations and DSS in HCC patients.

We investigated the genetic alterations of ANP32 family members. As shown in Figure 8(a), *ANP32E* mutations accounted for 5%, and the mutation form was mainly amplification. The mutation frequencies in *ANP32A* were 0.5%, and those in *ANP32B* were 0.2%. In addition, the relationship between genetic mutations of the ANP32 member and the prognosis of HCC patients was explored. HCC patients with ANP32 family mutations had worse OS than those without mutations (Figure 8(b)) (*P* < 0.01). Patients with ANP32 mutations tended to have worse DSS, but the difference was not statistically significant (Figure 8(c)) (*P* = 0.177).

## 4. Discussion

HCC, one of the most common tumors worldwide, is the leading cause of death in cancer patients. The primary challenge of treating HCC is achieving an early diagnosis. Thus, the identification of new promising biomarkers for diagnosing HCC and predicting disease progression, outcomes, and treatment effects is urgently needed. In this study, we focused on the diagnosis, prognosis, and possible biological functions of three members of the ANP32 family, namely, *ANP32A*, *ANP32B*, and *ANP32E*, in HCC.

*ANP32A* participates in many biological functions, such as regulating histone acetylation, transcription, DNA repair, and maintaining mRNA stability [17, 26, 27]. *ANP32A* plays different roles in different cancers. It has been reported that *ANP32A* can inhibit the progression of pancreatic cancer and lung cancer [28, 29]. However, certain studies have reported that *ANP32A* contributes to the development of cancers, such as leukemia, colorectal cancer, and glioma [14, 26, 30]. In a recent study, *ANP32A* was upregulated in HCC and promoted the proliferation and development of HCC by regulating the HMGA1/STAT3 pathway [17]. *ANP32B* plays an important role in the normal development of the body. Knockout of the *ANP32B* gene can cause mouse embryonic death [31]. Little research has focused on the correlation between *ANP32B* and tumors. Existing studies have shown that *ANP32B* has dual roles in different tumors. In acute leukemia, *ANP32B* can promote the apoptosis of leukemia cells by activating caspase-3 [11, 12]. A study on breast cancer showed that *ANP32B* is necessary not only for the normal development of the body but also for the growth of breast cancer cells [31]. In the only study on *ANP32B* and HCC, downregulation of *ANP32B* played an antiapoptotic effect, but upregulation of *ANP32B* did not lead to apoptosis of HCC cells [9]. *ANP32E* can promote cell proliferation in mammals and plays a role in DNA repair and the cell cycle [15]. Reports on *ANP32E* agreed that it is an oncogene. *ANP32E* contributes to the proliferation and migration of thyroid carcinoma cells by enhancing glycolysis mediated by AKT/mTOR/hk2 [10]. In triple-negative breast cancer, *ANP32E* can promote E2F1 transcription to promote G1/S transformation of TNBC cells, thereby inducing tumorigenesis [32]. In addition, it facilitates the progression of pancreatic cancer by regulating *β*-catenin [15]. However, no study has examined the association between *ANP32E* and HCC. Our functional enrichment analysis showed that ANP32 family members and their coexpressed genes are involved in proliferation-related processes such as DNA repair, the cell cycle, and RNA stabilization in HCC.

In our research, we found that the expression of the ANP32 family members *ANP32A*, *ANP32B*, and *ANP32E* was significantly upregulated in HCC at both the transcriptional and protein levels. The ROC curve showed that these three genes had outstanding diagnostic potential for HCC patients. High expression of the ANP32 family was connected to a higher pathologic stage and pathological grade. These results suggested that members of the ANP32 family can be used as indicators to assess the progression of HCC patients. Compared with primary HCC tissues, the expression of the ANP32 family in metastatic HCC tissue was significantly upregulated, indicating that they may participate in the progression and metastasis of HCC. Subsequent correlation analysis showed that the expression levels of *ANP32A*, *ANP32B*, and *ANP32E* in HCC were positively correlated with proliferation (Ki-67) and invasion (vimentin) markers, further indicating their role in the progression of HCC. It is worth noting that a previous study has shown that *ANP32A* plays a role in promoting cancer in HCC [17].

The prognostic value of ANP32 family members in cancer has been reported previously. *ANP32A* indicates a poor prognosis in HCC, acute myeloid leukemia, and glioma [14, 17, 33]. *ANP32B* is connected to a poor prognosis of breast cancer [16]. Higher *ANP32E* expression implies poor prognosis of pancreatic cancer patients and triple-negative breast cancer patients, and *ANP32E* can be used as an independent predictor of the outcome of triple-negative breast cancer [10, 15]. However, the prognostic role of *ANP32B* and *ANP32E* in HCC has not been previously reported, and whether *ANP32A* is an independent predictive factor for HCC has not been systematically studied. Here, we showed that the high expression levels of *ANP32A*, *ANP32B*, and *ANP32E* were related to shorter OS and DSS in HCC patients. Moreover, *ANP32B* and *ANP32E* can be used as independent predictors for OS and DSS.

Few reports have suggested the role of ANP32 family members in the immune response. Chemnitz et al. reported that *ANP32B* can play an immunomodulatory role in mice [34]. However, the role of ANP32 family members in the TME has not yet been fully studied. Here, we showed that the ANP32 family was related to immune cell infiltration and immune status in the HCC tumor microenvironment. *ANP32A*, *ANP32B*, and *ANP32E* are all highly expressed in C1 and C2 immune subtypes. Previous studies reported that C1 has elevated vascular gene expression, a high proliferation ratio and Th2-biased acquired immune infiltration, and C2 has the highest M1/M2 macrophage polarization, which seems to be consistent with our results of high immune cell infiltration in the ANP32 family member high expression group [24].

Recently, ICB therapy has become a promising treatment approach for patients with advanced HCC, but only a few patients are sensitive to ICB therapy [25, 35]. The TIDE score can predict the sensitivity of cancer patients to ICB treatment. Here, we showed that HCC patients with high *ANP32A*/B/E expression were more likely to be insensitive to ICB treatment. Thus, we explored the CellMiner database to find potential therapeutic drugs (Figure 8(g)). These drugs are FDA-approved and are used to treat other diseases. Our findings may provide new targets and possibilities for the treatment of HCC patients.

## 5. Conclusion

Our study suggested that ANP32 family expression was upregulated and had diagnostic potential in HCC. ANP32 family expression was associated with certain clinicopathological characteristics. *ANP32B* and *ANP32E* were independent prognostic biomarkers for OS and DSS in HCC patients. The high expression of the ANP32 family suggested that HCC patients had poor sensitivity to ICB treatment.

## Figures and Tables

**Table 1:** The association between *ANP32A* expression and clinical features of HCC patitents in TCGA cohort.

Characteristic	Low expression of *ANP32A*	High expression of *ANP32A*	*P*
*n*	187	187	
T stage, *n* (%)			0.188
T1	100 (27%)	83 (22.4%)	
T2	44 (11.9%)	51 (13.7%)	
T3	36 (9.7%)	44 (11.9%)	
T4	4 (1.1%)	9 (2.4%)	
N stage, *n* (%)			>0.999
N0	124 (48.1%)	130 (50.4%)	
N1	2 (0.8%)	2 (0.8%)	
M stage, *n* (%)			0.122
M0	134 (49.3%)	134 (49.3%)	
M1	0 (0%)	4 (1.5%)	
Pathologic stage, *n* (%)			0.264
Stage I	94 (26.9%)	79 (22.6%)	
Stage II	44 (12.6%)	43 (12.3%)	
Stage III	38 (10.9%)	47 (13.4%)	
Stage IV	1 (0.3%)	4 (1.1%)	
Tumor status, *n* (%)			0.122
Tumor free	109 (30.7%)	93 (26.2%)	
With tumor	69 (19.4%)	84 (23.7%)	
Gender, *n* (%)			0.047
Female	51 (13.6%)	70 (18.7%)	
Male	136 (36.4%)	117 (31.3%)	
Age, *n* (%)			0.034
≤60	78 (20.9%)	99 (26.5%)	
>60	109 (29.2%)	87 (23.3%)	
Weight, *n* (%)			0.126
≤70	86 (24.9%)	98 (28.3%)	
>70	90 (26%)	72 (20.8%)	
Height, *n* (%)			0.046
< 170	93 (27.3%)	108 (31.7%)	
≥170	81 (23.8%)	59 (17.3%)	
BMI, *n* (%)			0.470
≤25	86 (25.5%)	91 (27%)	
>25	85 (25.2%)	75 (22.3%)	
Histologic grade, *n* (%)			< 0.001
G1	39 (10.6%)	16 (4.3%)	
G2	97 (26.3%)	81 (22%)	
G3	46 (12.5%)	78 (21.1%)	
G4	3 (0.8%)	9 (2.4%)	
AFP (ng/ml), *n* (%)			< 0.001
≤400	131 (46.8%)	84 (30%)	
>400	16 (5.7%)	49 (17.5%)	
Vascular invasion, *n* (%)			0.019
No	119 (37.4%)	89 (28%)	
Yes	47 (14.8%)	63 (19.8%)	
Age, median (IQR)	62 (53, 69)	59 (51, 68)	0.171

**Table 2:** The association between *ANP32B* expression and clinical features of HCC patitents in TCGA cohort.

Characteristic	Low expression of *ANP32B*	High expression of *ANP32B*	*P*
*n*	187	187	
T stage, *n* (%)			0.005
T1	106 (28.6%)	77 (20.8%)	
T2	46 (12.4%)	49 (13.2%)	
T3	29 (7.8%)	51 (13.7%)	
T4	4 (1.1%)	9 (2.4%)	
N stage, *n* (%)			>0.999
N0	118 (45.7%)	136 (52.7%)	
N1	2 (0.8%)	2 (0.8%)	
M stage, *n* (%)			0.626
M0	126 (46.3%)	142 (52.2%)	
M1	1 (0.4%)	3 (1.1%)	
Pathologic stage, *n* (%)			0.024
Stage I	97 (27.7%)	76 (21.7%)	
Stage II	42 (12%)	45 (12.9%)	
Stage III	31 (8.9%)	54 (15.4%)	
Stage IV	2 (0.6%)	3 (0.9%)	
Tumor status, *n* (%)			0.036
Tumor free	111 (31.3%)	91 (25.6%)	
With tumor	66 (18.6%)	87 (24.5%)	
Gender, *n* (%)			0.185
Female	54 (14.4%)	67 (17.9%)	
Male	133 (35.6%)	120 (32.1%)	
Age, *n* (%)			0.133
≤60	81 (21.7%)	96 (25.7%)	
>60	106 (28.4%)	90 (24.1%)	
Weight, *n* (%)			0.010
≤70	80 (23.1%)	104 (30.1%)	
>70	94 (27.2%)	68 (19.7%)	
Height, *n* (%)			0.006
< 170	89 (26.1%)	112 (32.8%)	
≥170	84 (24.6%)	56 (16.4%)	
BMI, *n* (%)			0.024
≤25	79 (23.4%)	98 (29.1%)	
>25	92 (27.3%)	68 (20.2%)	
Histologic grade, *n* (%)			< 0.001
G1	35 (9.5%)	20 (5.4%)	
G2	102 (27.6%)	76 (20.6%)	
G3	47 (12.7%)	77 (20.9%)	
G4	1 (0.3%)	11 (3%)	
AFP (ng/ml), *n* (%)			< 0.001
≤400	126 (45%)	89 (31.8%)	
>400	13 (4.6%)	52 (18.6%)	
Vascular invasion, *n* (%)			0.147
No	110 (34.6%)	98 (30.8%)	
Yes	48 (15.1%)	62 (19.5%)	
Age, median (IQR)	62 (54, 69)	60 (51, 69)	0.141

**Table 3:** The association between *ANP32E* expression and clinical features of HCC patitents in TCGA cohort.

Characteristic	Low expression of *ANP32E*	High expression of *ANP32E*	*P*
*n*	187	187	
T stage, *n* (%)			0.056
T1	104 (28%)	79 (21.3%)	
T2	43 (11.6%)	52 (14%)	
T3	32 (8.6%)	48 (12.9%)	
T4	6 (1.6%)	7 (1.9%)	
N stage, *n* (%)			>0.999
N0	122 (47.3%)	132 (51.2%)	
N1	2 (0.8%)	2 (0.8%)	
M stage, *n* (%)			0.622
M0	133 (48.9%)	135 (49.6%)	
M1	1 (0.4%)	3 (1.1%)	
Pathologic stage, *n* (%)			0.055
Stage I	99 (28.3%)	74 (21.1%)	
Stage II	42 (12%)	45 (12.9%)	
Stage III	34 (9.7%)	51 (14.6%)	
Stage IV	2 (0.6%)	3 (0.9%)	
Tumor status, *n* (%)			0.204
Tumor free	106 (29.9%)	96 (27%)	
With tumor	69 (19.4%)	84 (23.7%)	
Gender, *n* (%)			0.122
Female	53 (14.2%)	68 (18.2%)	
Male	134 (35.8%)	119 (31.8%)	
Age, *n* (%)			0.196
≤60	82 (22%)	95 (25.5%)	
>60	105 (28.2%)	91 (24.4%)	
Weight, *n* (%)			0.050
≤70	84 (24.3%)	100 (28.9%)	
>70	92 (26.6%)	70 (20.2%)	
Height, *n* (%)			0.415
< 170	96 (28.2%)	105 (30.8%)	
≥170	74 (21.7%)	66 (19.4%)	
BMI, *n* (%)			0.409
≤25	85 (25.2%)	92 (27.3%)	
>25	85 (25.2%)	75 (22.3%)	
Histologic grade, *n* (%)			0.002
G1	36 (9.8%)	19 (5.1%)	
G2	97 (26.3%)	81 (22%)	
G3	46 (12.5%)	78 (21.1%)	
G4	6 (1.6%)	6 (1.6%)	
AFP (ng/ml), *n* (%)			< 0.001
≤400	122 (43.6%)	93 (33.2%)	
>400	19 (6.8%)	46 (16.4%)	
Vascular invasion, *n* (%)			0.249
No	112 (35.2%)	96 (30.2%)	
Yes	51 (16%)	59 (18.6%)	
Age, median (IQR)	63 (53, 70)	60 (51, 67.75)	0.031

**Table 4:** Univariate and multivariate Cox regression analyses of selected variables on OS.

Characteristics	Total (N)	Univariate analysis		Multivariate analysis	
		Hazard ratio (95% CI)	*P* value	Hazard ratio (95% CI)	*P* value
*ANP32A*	373	1.534 (1.073-2.192)	0.019	0.791 (0.484-1.291)	0.348
*ANP32B*	373	1.776 (1.351-2.334)	<0.001	1.745 (1.212-2.514)	0.003
*ANP32E*	373	1.636 (1.279-2.092)	<0.001	1.350 (1.017-1.793)	0.038
Pathologic stage	349				
Stage I	173	Reference			
Stage II	86	1.417 (0.868-2.312)	0.164	1.190 (0.723-1.960)	0.494
Stage III	85	2.734 (1.792-4.172)	<0.001	2.253 (1.458-3.484)	<0.001
Stage IV	5	5.597 (1.726-18.148)	0.004	7.589 (2.195-26.242)	0.001
Gender	373				
Male	252	Reference			
Female	121	1.261 (0.885-1.796)	0.200		
Histologic grade	368				
G1	55	Reference			
G2	178	1.162 (0.686-1.969)	0.576		
G3	123	1.185 (0.683-2.057)	0.545		
G4	12	1.681 (0.621-4.549)	0.307		
Age	373				
≤60	177	Reference			
>60	196	1.205 (0.850-1.708)	0.295		

**Table 5:** Univariate and multivariate Cox regression analyses of selected variables on DSS.

Characteristics	Total (N)	Univariate analysis		Multivariate analysis	
		Hazard ratio (95% CI)	*P* value	Hazard ratio (95% CI)	*P* value
*ANP32A*	365	1.539 (0.975-2.430)	0.064		
*ANP32B*	365	1.637 (1.146-2.337)	0.007	1.547 (1.019-2.347)	0.040
*ANP32E*	365	1.840 (1.338-2.530)	<0.001	1.495 (1.035-2.159)	0.032
Pathologic stage	341				
Stage I	170	Reference			
Stage II	84	1.561 (0.776-3.141)	0.212	1.306 (0.642-2.655)	0.461
Stage III	83	4.288 (2.438-7.543)	<0.001	3.669 (2.066-6.515)	<0.001
Stage IV	4	9.369 (2.171-40.437)	0.003	11.499 (2.640-50.092)	0.001
Gender	365				
Male	247	Reference			
Female	118	1.230 (0.780-1.937)	0.373		
Histologic grade	360				
G1	55	Reference			
G2	172	1.177 (0.599-2.314)	0.636		
G3	121	1.232 (0.610-2.486)	0.561		
G4	12	1.181 (0.260-5.361)	0.829		
Age	365				
≤60	174	Reference			
>60	191	0.846 (0.543-1.317)	0.458		

## Data Availability

The databases mentioned in the study are publicly and freely available.
